# Supplementary material for: Estimating the total variance explained by whole-brain imaging for zero-inflated outcomes
Source: Commun Biol. 2024 Jul 9;7:836. doi: 10.1038/s42003-024-06504-y (PMC11233705; doi:10.1038/s42003-024-06504-y)
Supplement: Supplementary file 2 — Supplementary Information [file 42003_2024_6504_MOESM2_ESM.pdf]

Supplementary Materials: Estimating the Total Variance Explained by Whole-Brain Imaging for Zero-inflated Outcomes

Junting Ren[1†], Robert Loughnan[2,3], Bohan Xu[3], Wesley K. Thompson[3] and Chun Chieh Fan[3,4†]

[1]Division of Biostatistics, Herbert Wertheim School of Public Health and Human Longevity Science, University of California San Diego, 9500 Gilman Street, La Jolla, 92093, CA, USA.
[2]Center for Human Development, University of California San Diego, 9500 Gilman Drive, La Jolla, 92093, CA, USA.
[3]Center for Population Neuroscience and Genetics, Laureate Institute for Brain Research, 6655 S Yale Ave, Tulsa, 74136, OK, USA.
[4]Department of Radiology, School of Medicine, University of California San Diego, 9500 Gilman Drive, La Jolla, 92093, CA, USA.

[†]Corresponding authors

Supplementary Note 1 Variational Inference Loss Derivation and Optimization

Supplementary Note 1.1 Expectation of the data likelihood

We detail the derivation for the expectation of the data likelihood over the variational distribution (Equation 12 in the main text). Due to the truncation

of the observed phenotype z, the log data likelihood for $z = 0$ is

$$
\begin{aligned}
&\log \mathbb{P}(\boldsymbol{z} \mid \tilde{\boldsymbol{\beta}}, \boldsymbol{\delta}, \pi, \sigma) \\
&= \sum_{i=1}^{n} \log \mathbb{P}(y_i \leq 0) \\
&= \sum_{i=1}^{n} \log \mathbb{P}\left(\sum_{j=1}^{p} x_{ij} \tilde{\beta}_j \delta_j + \beta_0 + \epsilon_i \leq 0 \right) \\
&= \sum_{i=1}^{n} \log \mathbb{P}\left(\frac{\epsilon_i}{\sigma} \leq -\frac{1}{\sigma} (\sum_{j=1}^{p} x_{ij} \tilde{\beta}_j \delta_j + \beta_0) \right) \\
&= \sum_{i=1}^{n} \log \Phi\left(-\frac{1}{\sigma} (\sum_{j=1}^{p} x_{ij} \tilde{\beta}_j \delta_j + \beta_0) \right)
\end{aligned}
\tag{S1}
$$

where Φ is the cumulative distribution function for standard normal distribution. The log data likelihood when $z > 0$ is given by

$$
\begin{aligned}
&\log \mathbb{P}(\boldsymbol{z} \mid \tilde{\boldsymbol{\beta}}, \boldsymbol{\delta}, \pi, \sigma) \\
&= \sum_{i=1}^{n} \left[-\frac{1}{2} \log(2\sigma^2) - \frac{(z_i - \sum_{j=1}^{p} x_{ij} \tilde{\beta}_j \delta_j - \beta_0)^2}{2\sigma^2} \right] \\
&= -\frac{n}{2} \log(2\sigma^2) - \frac{\sum_{i=1}^{n} (z_i - \sum_{j=1}^{p} x_{ij} \tilde{\beta}_j \delta_j - \beta_0)^2}{2\sigma^2}
\end{aligned}
\tag{S2}
$$

The variational parameters that need to be taken expectation of are σ^2, $\tilde{\boldsymbol{\beta}}, \boldsymbol{\delta}$. When $z = 0$, we directly plug in the current estimate μ_{β_j} in place of $\tilde{\beta}_j$, π_{β_j} in place of δ_j, and $\sqrt{\exp(b_3 + b_4)}$ (mode of the standard deviation variational distribution) in place of σ into Equation S1 as approximation to the expectation of Equation S1.

For the expectation of the log data likelihood when $z > 0$, it is possible to calculate the exact value. Due to the factorization of the approximation distribution, we can first take the expectation with respect to σ,

$$
\begin{aligned}
&\mathbb{E}_{\sigma^2 \sim q}[\log \mathbb{P}(\boldsymbol{y} \mid \tilde{\boldsymbol{\beta}}, \boldsymbol{\delta}, \pi, \sigma)] \\
&= -\frac{n}{2} b_3 - \frac{1}{2} \exp(-b_3 + b_4/2) \sum_{i=1}^{n} (z_i - \sum_{j=1}^{p} x_{ij} \tilde{\beta}_j \delta_j - \beta_0)^2
\end{aligned}
\tag{S3}
$$

As for $\tilde{\boldsymbol{\beta}}, \boldsymbol{\delta}$, we need to expand $(z_i - \sum_{j=1}^{p} x_{ij} \tilde{\beta}_j \delta_j - \beta_0)^2$ before taking the expectation. We use the Gumbel-softmax [1] to sample δ_j and reparametrization trick [2] to sample $\tilde{\beta}_j$ so that there will be gradients for the respective parameters using Monte Carlo integration. The best temperature for Gumbel-softmax is 1. During simulation experimentation for the Monte

Carlo integration model, it was observe that even by taking more samples for the integration, the performance did not improve. From further investigation, this is because simply by plugging in μ_{β_j} and π_{β_j} in place of β_j and δ_j, the only difference between the true expectation and the plug-in approximation is on the quadratic term, where the true expectation contains $\sum_{j=1}^{p} x_{ij}^2(\mu_{\beta_j}^2 + \sigma^2)\pi_{\beta_j}$ comparing the approximation $\sum_{j=1}^{p} x_{ij}^2 \mu_{\beta_j}^2 \pi_{\beta_j}^2$ for each i. By taking the difference, we can obtain the true expectation. The true expectation of the term $(z_i - \sum_{j=1}^{p} x_{ij}\tilde{\beta}_j\delta_j - \beta_0)^2$ is:

$$\sum_{i=1}^{n} \Big[z_i^2 + \beta_0^2 + \sum_{j=1}^{p} x_{ij}(\mu_{\beta_j}^2 + \sigma_{\beta_j}^2)\pi_{\beta_j} - 2z_i(\sum_{j=1}^{p} x_{ij}\mu_{\beta_j}\pi_{\beta_j}) \\ - 2z_i\beta_0 + 2\beta_0(\sum_{j=1}^{p} x_{ij}\mu_{\beta_j}\pi_{\beta_j}) + 2\sum_{k<l} x_{ik}x_{il}\mu_{\beta_k}\mu_{\beta_l}\pi_{\beta_k}\pi_{\beta_l}\Big] \tag{S4}$$

Note that this is exactly the same as the variational inference posterior if β_j and δ_j are independent since the conditional dependency is not introduced in the data likelihood. Therefore, in the final algorithm, we used the true expectation of the data likelihood to optimize over instead of the Gumbel-softmax approximation version.

Supplementary Note 1.2 Expectation of the prior likelihood

We detail the derivation for expectation of the prior likelihood over the variational distribution. Expanding the inside of Equation 13 in the main text,

$$\mathbb{P}(\tilde{\boldsymbol{\beta}}, \boldsymbol{\delta}, \pi, \sigma) \\ = \left[\prod_{j=1}^{p} \pi^{\delta_j}(1-\pi)^{1-\delta_j} \frac{1}{\sqrt{2\sigma_\beta^2}} \exp\left(-\frac{\tilde{\beta}_j^2}{2\sigma_\beta^2}\right)\right] \tag{S5}$$

Therefore, taking the log, we have

$$\log \mathbb{P}(\tilde{\boldsymbol{\beta}}, \boldsymbol{\delta}, \pi, \sigma) \\ = \log(\pi)\sum_{j=1}^{p} \delta_j + \log(1-\pi)\sum_{j=1}^{p}(1-\delta_j) - 0.5p\log(2\sigma_\beta^2) \\ - \frac{\sum_{j=1}^{p} \tilde{\beta}_j^2}{2\sigma_\beta^2} \tag{S6}$$

Now, we know

$$\mathbb{E}_{\pi\sim\phi}(\log(\pi)) = \psi(c) - \psi(c+d)$$

$$\mathbb{E}_{\pi\sim\phi}(\log(1-\pi)) = \psi(d) - \psi(c+d)$$
$$\mathbb{E}_{\tilde{\beta}_j\sim\phi}(\tilde{\beta}_j^2) = \mathbb{E}\left(\mathbb{E}(\tilde{\beta}_j^2 \mid \delta_j)\right) = \mu_{\beta_j}^2\pi_j + \sigma_{\beta_j}^2\pi_j + (1-\pi_j)\sigma_\beta^2 \quad \text{(S7)}$$

where ψ is the digamma function. From linearity of the expectation, we can obtain

$$\mathbb{E}_\phi\left[\log\mathbb{P}(\tilde{\boldsymbol{\beta}},\boldsymbol{\delta},\pi,\sigma)\right]$$
$$= \mathbb{E}[\log(\pi)]\sum_{j=1}^{p}\pi_{\beta_j} + \mathbb{E}[\log(1-\pi)]\sum_{j=1}^{p}(1-\pi_{\beta_j}) - 0.5p\log(2\sigma_\beta^2)$$
$$- \frac{\sum_{j=1}^{p}\mathbb{E}[\tilde{\beta}_j^2]}{2\sigma_\beta^2} \quad \text{(S8)}$$

Supplementary Note 1.3 Entropy

We detail the derivation for Entropy term (Equation 14 in the main text). Since the variational approximation distributions are independent implying that they can be factorized, we consider the entropy for $\tilde{\boldsymbol{\beta}},\boldsymbol{\delta}$ first:

$$\mathbb{E}_{q(\tilde{\boldsymbol{\beta}},\boldsymbol{\delta})}\left[-\log q(\tilde{\boldsymbol{\beta}},\boldsymbol{\delta})\right]$$
$$= -\sum_{j=1}^{p}\left[\int(1-\pi_j)\mathcal{N}\left(\beta_j \mid 0,\sigma_\beta^2\right)\log\left[(1-\pi_j)\mathcal{N}\left(\beta_j \mid 0,\sigma_\beta^2\right)\right]d\beta_j\right.$$
$$\left.+\int\pi_j\mathcal{N}\left(\beta_j \mid \mu_{\beta_j},\sigma_{\beta_j}^2\right)\log\left[\pi_j\mathcal{N}\left(\beta_j \mid \mu_{\beta_j},\sigma_{\beta_j}^2\right)\right]d\beta_j\right]$$
$$= \sum_{j=1}^{p}\left[-(1-\pi_j)\log(1-\pi_j) - \pi_j\log(\pi_j)\right.$$
$$\left.+\frac{1}{2}(1-\pi_j)\log(2\pi e\sigma_\beta^2) + \frac{1}{2}\pi_j\log(2\pi e\sigma_{\beta_j}^2)\right] \quad \text{(S9)}$$

The entropy for π is

$$\mathbb{E}_{q(\pi)}\left[-\log q(\pi)\right]$$
$$= \log\left(\frac{\Gamma(a_3)\Gamma(a_4)}{\Gamma(a_3+a_4)}\right) - (a_3-1)\psi(a_3)$$
$$-(a_4-1)\psi(a_4) + (a_3+a_4-2)\psi(a_3+a_4) \quad \text{(S10)}$$

where Γ is the gamma function. The entropy for σ is

$$\mathbb{E}_{q(\sigma)}\left[-\log q(\sigma)\right]$$

$$\propto \log\left(\sqrt{b_4}\exp(b_3 + 0.5)\right) \tag{S11}$$

Supplementary Note 1.4 Variational Inference Model Optimization

After deriving the explicit form of Equation 12, 13 and 14 in the main text, we obtain the model loss (ELBO, sum of the three equations) that we want to minimize over. Majority of the parameters that needs to be optimized are initialized randomly using either standard Gaussian distribution or Uniform distribution [3]. For the posterior approximating Beta distribution for π, the parameters a_3 and a_4 are initialized with 1.1. For the posterior approximating LogNormal distribution for σ^2, the parameters b_3 and b_4 are initialized at 10.0 and 0.1, respectively.

We used the Adam optimizer [4] with learning rate of 0.5, with betas equal to (0.9, 0.999). An exponential learning rate scheduler is used with decay multiplicative factor 0.8 for every 1000 epochs. Early stop is implemented whenever the difference between the current loss and minimum loss is less than 1% for 200 epochs, with a max of total training epochs of 20000. Bayesian variational inference inherently guards against overfitting, so there is no need for a validation set to be used for early stopping. In this context, early stopping has been implemented specifically to expedite the training process.

Supplementary Note 2 Markov Chain Monte Carlo Algorithm

We outlined the MCMC algorithm in the main text (Algorithm 1 in the main text), but the HMC and Gibbs sampling steps are not explained. Therefore, we expand the details for the two sampling algorithms. First, we simply the notation by denoting the all the continuous ($\tilde{\boldsymbol{\beta}}, \pi,$ and σ) as $\boldsymbol{\theta}$, and the log of the posterior distribution as $\mathcal{L}$.

Algorithm 1 Hamiltonian Monte Carlo sampling algorithm

Require: $\boldsymbol{\theta}^0, \epsilon, L, M$, $\mathcal{L}$ conditioned on $\boldsymbol{\delta}, \boldsymbol{z}, \boldsymbol{X}$.
for $m = 1$ to M **do**
  Sample $\boldsymbol{r}^0 \sim \mathcal{N}(0, I)$.
  Set $\boldsymbol{\theta}^m \leftarrow \boldsymbol{\theta}^{m-1}, \tilde{\boldsymbol{\theta}} \leftarrow \boldsymbol{\theta}^{m-1}, \tilde{\boldsymbol{r}} \leftarrow \boldsymbol{r}^0$.
  for $i = 1$ to L **do**
    Set $\tilde{\boldsymbol{\theta}}, \tilde{\boldsymbol{r}} \leftarrow \text{Leapfrog}(\tilde{\boldsymbol{\theta}}, \tilde{\boldsymbol{r}}, \epsilon)$.
  end for
  With probability $\alpha = \min\left\{1, \frac{\exp\left\{\mathcal{L}(\tilde{\boldsymbol{\theta}}) - \frac{1}{2}\tilde{\boldsymbol{r}}\cdot\tilde{\boldsymbol{r}}\right\}}{\exp\left\{\mathcal{L}\left(\boldsymbol{\theta}^{m-1}\right) - \frac{1}{2}\boldsymbol{r}^0\cdot\boldsymbol{r}^0\right\}}\right\}$, set $\boldsymbol{\theta}^m \leftarrow \tilde{\boldsymbol{\theta}}$.
end for
function Leapfrog$(\boldsymbol{\theta}, \boldsymbol{r}, \epsilon)$
Set $\tilde{\boldsymbol{r}} \leftarrow \boldsymbol{r} + (\epsilon/2)\nabla_\theta \mathcal{L}(\boldsymbol{\theta})$.
Set $\tilde{\boldsymbol{\theta}} \leftarrow \theta + \epsilon\tilde{\boldsymbol{r}}$.
Set $\tilde{\boldsymbol{r}} \leftarrow \tilde{\boldsymbol{r}} + (\epsilon/2)\nabla_\theta \mathcal{L}(\tilde{\boldsymbol{\theta}})$.
return $\tilde{\boldsymbol{\theta}}, \tilde{\boldsymbol{r}}$.

Algorithm 2 Gibbs Monte Carlo sampling algorithm

Require: $\boldsymbol{\delta}^0, \boldsymbol{\theta}$, p is the number of features, $\mathcal{L}$ conditioned on $\boldsymbol{\theta}, \boldsymbol{z}, \boldsymbol{X}$.
Require: $\tilde{\boldsymbol{\delta}} = \{\}$
for $j = 1$ to p **do**
  Sample $\tilde{\delta}_j \sim \text{Bernoulli}\left(\frac{\exp[\mathcal{L}(\delta_j=1, \boldsymbol{\delta}^0_{-j})]}{\exp[\mathcal{L}(\delta_j=1, \boldsymbol{\delta}^0_{-j})] + \exp[\mathcal{L}(\delta_j=0, \boldsymbol{\delta}^0_{-j})]}\right)$
  Append $\tilde{\delta}_j$ to $\tilde{\boldsymbol{\delta}}$
end for
return $\tilde{\boldsymbol{\delta}}$

Supplementary Table 1 Number of observations and features for data application

modality	N	p
Resting state MRI	9501	416
Task functional MRI	8893	885
Structural MRI	9426	1186
Diffusion tensor images	9454	2367
Restricted spectrum images	9455	1140

References

[1] Eric Jang, Shixiang Gu, and Ben Poole. Categorical reparameterization with gumbel-softmax. *arXiv preprint arXiv:1611.01144*, 2016.

[2] Durk P Kingma, Tim Salimans, and Max Welling. Variational dropout and the local reparameterization trick. *Advances in neural information processing systems*, 28, 2015.

[3] Kaiming He, Xiangyu Zhang, Shaoqing Ren, and Jian Sun. Delving deep into rectifiers: Surpassing human-level performance on imagenet classification. In *Proceedings of the IEEE international conference on computer vision*, pages 1026–1034, 2015.

[4] Diederik P Kingma and Jimmy Ba. Adam: A method for stochastic optimization. *arXiv preprint arXiv:1412.6980*, 2014.
