# Supplementary material for: Estimating the total variance explained by whole-brain imaging for zero-inflated outcomes
Source: Commun Biol. 2024 Jul 9;7:836. doi: 10.1038/s42003-024-06504-y (PMC11233705; doi:10.1038/s42003-024-06504-y)
Supplement: Supplementary file 3 — Description of Additional Supplementary Files [file 42003_2024_6504_MOESM3_ESM.pdf]

Description of Additional Supplementary Files

File name: Supplementary Data 1

Description: The source data for the Figure 2a and 3a.

File name: Supplementary Data 2

Description: The source data for the Figure 2b and 3b.

File name: Supplementary Data 3

Description: The source data for the Figure 4.

File name: Supplementary Data 4

Description: The source data for the Figure 5.

File name: Supplementary Data 5

Description: Imaging variables and their corresponding categories
