# Supplementary material for: Estimating the total variance explained by whole-brain imaging for zero-inflated outcomes
Source: Commun Biol. 2024 Jul 9;7:836. doi: 10.1038/s42003-024-06504-y (PMC11233705; doi:10.1038/s42003-024-06504-y)
Supplement: Supplementary file 5 — Reporting Summary [file 42003_2024_6504_MOESM5_ESM.pdf]

nature portfolio

Corresponding author(s): Chun Chieh Fan
Last updated by author(s): 06/04/2024

# Reporting Summary

Nature Portfolio wishes to improve the reproducibility of the work that we publish. This form provides structure for consistency and transparency in reporting. For further information on Nature Portfolio policies, see our Editorial Policies and the Editorial Policy Checklist.

## Statistics

For all statistical analyses, confirm that the following items are present in the figure legend, table legend, main text, or Methods section.

| n/a | Confirmed | |
|---|---|---|
| ☐ | ☒ | The exact sample size ($n$) for each experimental group/condition, given as a discrete number and unit of measurement |
| ☐ | ☒ | A statement on whether measurements were taken from distinct samples or whether the same sample was measured repeatedly |
| ☐ | ☒ | The statistical test(s) used AND whether they are one- or two-sided<br>*Only common tests should be described solely by name; describe more complex techniques in the Methods section.* |
| ☐ | ☒ | A description of all covariates tested |
| ☐ | ☒ | A description of any assumptions or corrections, such as tests of normality and adjustment for multiple comparisons |
| ☐ | ☒ | A full description of the statistical parameters including central tendency (e.g. means) or other basic estimates (e.g. regression coefficient) AND variation (e.g. standard deviation) or associated estimates of uncertainty (e.g. confidence intervals) |
| ☐ | ☒ | For null hypothesis testing, the test statistic (e.g. $F$, $t$, $r$) with confidence intervals, effect sizes, degrees of freedom and $P$ value noted<br>*Give P values as exact values whenever suitable.* |
| ☐ | ☒ | For Bayesian analysis, information on the choice of priors and Markov chain Monte Carlo settings |
| ☐ | ☒ | For hierarchical and complex designs, identification of the appropriate level for tests and full reporting of outcomes |
| ☐ | ☒ | Estimates of effect sizes (e.g. Cohen's $d$, Pearson's $r$), indicating how they were calculated |

*Our web collection on statistics for biologists contains articles on many of the points above.*

## Software and code

Policy information about availability of computer code

| | |
|---|---|
| Data collection | The data is provided by ABCD Study from their public annual data release. Therefore, no code for data collection is used here. |
| Data analysis | The code associated with this research is available on GitHub at https://github.com/junting-ren/ZIV. This repository contains all necessary code and instructions to replicate the analyses described in this paper. |

For manuscripts utilizing custom algorithms or software that are central to the research but not yet described in published literature, software must be made available to editors and reviewers. We strongly encourage code deposition in a community repository (e.g. GitHub). See the Nature Portfolio guidelines for submitting code & software for further information.

## Data

Policy information about availability of data

All manuscripts must include a data availability statement. This statement should provide the following information, where applicable:

- Accession codes, unique identifiers, or web links for publicly available datasets
- A description of any restrictions on data availability
- For clinical datasets or third party data, please ensure that the statement adheres to our policy

ABCD data are released publicly on an annual basis via the National Institute of Mental Health Data Archive (NDA, \url{https://data-archive.nimh.nih.gov/abcd}).

More details about the study can be found at \url{https://abcdstudy.org/}. In this application, we used data from the ABCD 4.0 National Data Archive release (NDAR DOI:10.15154/1523041). We also provide all the relevant result outputs as supplementary tables.

## Research involving human participants, their data, or biological material

Policy information about studies with human participants or human data. See also policy information about sex, gender (identity/presentation), and sexual orientation and race, ethnicity and racism.

| | |
|---|---|
| Reporting on sex and gender | The biological sex is one of the covariates in the models  we used for empirical analysis to avoid potential bias. |
| Reporting on race, ethnicity, or other socially relevant groupings | Genetic principal components, that can capture potential population structure, are included in the model for the empirical analysis to avoid potential bias. |
| Population characteristics | In addition to biological sex, population structure, we also controls for age, scanner info, and family relatedness. |
| Recruitment | ABCD study has multi-site school-based recruitment design, yielding a demographic distribution similar to the general distribution in the United States. We did not impose any additional selection criteria except the data availability from the public data release. |
| Ethics oversight | ABCD Study is governed by the ABCD IRB review, which is a multi-institute effort funded by NIH. |

Note that full information on the approval of the study protocol must also be provided in the manuscript.

# Field-specific reporting

Please select the one below that is the best fit for your research. If you are not sure, read the appropriate sections before making your selection.

☐ Life sciences ☒ Behavioural & social sciences ☐ Ecological, evolutionary & environmental sciences

For a reference copy of the document with all sections, see nature.com/documents/nr-reporting-summary-flat.pdf

# Behavioural & social sciences study design

All studies must disclose on these points even when the disclosure is negative.

| | |
|---|---|
| Study description | The data is quantitative, obtained from a multi-site neuorimaging study of youth in United States. |
| Research sample | The research samples are children with age 9-10 from United States. The demographic characteristics are matched with the overall distribution in the United States, based on the American Census. |
| Sampling strategy | ABCD ascertain their samples through school recruitment design with additional diversity criteria to assist the representativeness of the sample. |
| Data collection | The data includes MRI scans and behavioral questionnaires through onsite assessment. |
| Timing | The MRI is collected every other year and the behavioral questionnaires are collected annually. ABCD Study is an ongoing longitudinal study. |
| Data exclusions | Data that fails the imaging quality control and miss behavioral assessments are excluded from the empirical analyses. |
| Non-participation | ABCD Study retains participation rate above 90%. |
| Randomization | No randomization is involved. |

# Reporting for specific materials, systems and methods

We require information from authors about some types of materials, experimental systems and methods used in many studies. Here, indicate whether each material, system or method listed is relevant to your study. If you are not sure if a list item applies to your research, read the appropriate section before selecting a response.

## Materials & experimental systems

| n/a | Involved in the study |
|---|---|
| ☒ | ☐ Antibodies |
| ☒ | ☐ Eukaryotic cell lines |
| ☒ | ☐ Palaeontology and archaeology |
| ☒ | ☐ Animals and other organisms |
| ☒ | ☐ Clinical data |
| ☒ | ☐ Dual use research of concern |
| ☒ | ☐ Plants |

## Methods

| n/a | Involved in the study |
|---|---|
| ☒ | ☐ ChIP-seq |
| ☒ | ☐ Flow cytometry |
| ☐ | ☒ MRI-based neuroimaging |

# Plants

Seed stocks: *Report on the source of all seed stocks or other plant material used. If applicable, state the seed stock centre and catalogue number. If plant specimens were collected from the field, describe the collection location, date and sampling procedures.*

Novel plant genotypes: *Describe the methods by which all novel plant genotypes were produced. This includes those generated by transgenic approaches, gene editing, chemical/radiation-based mutagenesis and hybridization. For transgenic lines, describe the transformation method, the number of independent lines analyzed and the generation upon which experiments were performed. For gene-edited lines, describe the editor used, the endogenous sequence targeted for editing, the targeting guide RNA sequence (if applicable) and how the editor was applied.*

Authentication: *Describe any authentication procedures for each seed stock used or novel genotype generated. Describe any experiments used to assess the effect of a mutation and, where applicable, how potential secondary effects (e.g. second site T-DNA insertions, mosiacism, off-target gene editing) were examined.*

# Magnetic resonance imaging

## Experimental design

Design type: Multi-modal MRI of human brain, including structural T1, diffusion imaging, resting state fMRI, and task fMRI

Design specifications: As released by ABCD Study, detailed in the study protocol.

Behavioral performance measures: As released by ABCD Study, detailed in the study protocol.

## Acquisition

Imaging type(s): Structural T1, diffusion, resting state, and task fMRI

Field strength: 3T

Sequence & imaging parameters: See ABCD Study imaging protocol as cited.

Area of acquisition: whole brain

Diffusion MRI: ☒ Used ☐ Not used

Parameters: *Specify # of directions, b-values, whether single shell or multi-shell, and if cardiac gating was used.*

## Preprocessing

Preprocessing software: See ABCD preprocessing paper as cited in the method.

Normalization: *If data were normalized/standardized, describe the approach(es): specify linear or non-linear and define image types used for transformation OR indicate that data were not normalized and explain rationale for lack of normalization.*

Normalization template: *Describe the template used for normalization/transformation, specifying subject space or group standardized space (e.g. original Talairach, MNI305, ICBM152) OR indicate that the data were not normalized.*

Noise and artifact removal: *Describe your procedure(s) for artifact and structured noise removal, specifying motion parameters, tissue signals and physiological signals (heart rate, respiration).*

Volume censoring: *Define your software and/or method and criteria for volume censoring, and state the extent of such censoring.*

## Statistical modeling & inference

Model type and settings: We developed the algorithm, ZIV, and all are described in the main text.

Effect(s) tested: correlations between behavior and imaging feature.

Specify type of analysis: ☒ Whole brain ☐ ROI-based ☐ Both

Statistic type for inference (See Eklund et al. 2016): experiment-wise estimation.

Correction: Bayesian analysis with credible intervals

## Models & analysis

| n/a | Involved in the study |
| --- | --- |
| ☒ | ☐ Functional and/or effective connectivity |
| ☒ | ☐ Graph analysis |
| ☐ | ☒ Multivariate modeling or predictive analysis |

Multivariate modeling and predictive analysis: Mixed effects regression method with zero inflated non-normal outcomes.
